# Effects of Surface Morphology and Type of Cross-Linking of Chitosan-Pectin Microspheres on Their Degree of Swelling and Favipiravir Release Behavior

**DOI:** 10.3390/polym15153173

**Published:** 2023-07-26

**Authors:** Amer Rashid Hameed, Hatem Majdoub, Fawzi Habeeb Jabrail

**Affiliations:** 1 The State Company for Drugs Industry and Medical Appliances, Samaraa 34010, Iraq; chem.ammer72@gmail.com; 2 Laboratory of Interfaces and Advanced Materials, Faculty of Science of Monastir, University of Monastir, Monastir 5000, Tunisia; hatemmajdoub.fsm@gmail.com; 3 Polymer Research Laboratory, Department of Chemistry, Collage of Science, University of Mosul, Mosul 41002, Iraq

**Keywords:** Favipiravir, pomegranate peels, chitosan, pectin, controlled drug release, gluteraldehyde, sodium hexametaphosphate

## Abstract

The cross-linked microspheres were prepared and loaded with Favipiravir SARS-CoV-2 antiviral drug, by copolymerization of chitosan (CS) with a polysaccharide extracted from fresh pomegranate peels. Moreover, glutaraldehyde (Glu) has been used as a chemical cross-linker and sodium hexametaphosphate (SHMP) as a physical cross-linker. The extracted polysaccharide was analyzed, and different techniques have been used. The analyses lead to the conclusion that it is pectin. The surface morphology of the prepared microspheres was studied using a scanning electron microscope, where the size and shape factor (S) of the Glu microspheres showed high values (74.27 μm) and (0.852), respectively, meaning their surfaces tend to be rough, whereas the SHMP microspheres showed a smaller size particle (20.47 μm) and a smaller shape factor (0.748), which gives an indication that the SHMP microspheres have smooth surfaces. The swelling studies have shown that Glu microspheres have a higher degree of swelling, which means SHMP microspheres are more compact. The prepared microspheres have shown a higher loading percentage of Favipiravir antiviral drug in SHMP microspheres (37% *w*/*w*) in comparison with Glu microspheres (35% *w*/*w*), where the electrostatic interaction between the Favipiravir ions and SHMP anions helps for more loading. The microspheres prepared under different types of cross-linking have shown initial burst release of Favipiravir, followed by a step of controlled release for a certain period of time, whose period depends on the pH of the release medium. Both Glu and SHMP cross-linked microspheres have shown high controlled release times in buffered release solutions at pH = 7.4 and for shorter periods at pH = 1.3 and pH = 9.4, which may be related to the type of electrostatic interactions between drug and polymer systems and their reactions with release solution ions.

## 1. Introduction 

**Figure 1:**
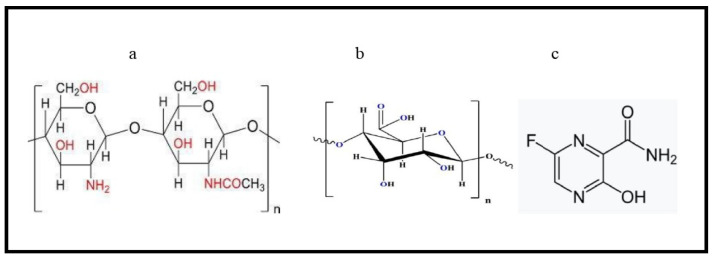
Chemical structure of (**a**) chitosan, (**b**) Pectin, and (**c**) Favipiravir.

Recently, some polysaccharides with biomedical properties have received great attention for sustained delivery of drugs [1]. Natural polysaccharides, pectin and chitosan in particular, have expanded biological properties and a wide range of applications in pharmacology [2,3]. Polymers have been improved to have a technique that adds effort to the development of different technologies such as bioengineering, biotechnology, and medicine [4,5,6]. Drug delivery systems were early designs for delivering medication to a certain target inside a patient to increase the drug concentration in that part and prevent any interactions [7]. Biomaterials, or polymers, showed the ability to modify the pharmacokinetics of the drug [8]. The selection of polymers could play a significant role in designing a drug delivery system with a controlled profile and known physicochemical properties [9]. Polysaccharides are one type of biomaterial used in drug delivery systems and have formulations suitable for drug delivery. Chitosan and pectin are polysaccharides of cationic and anionic structures, respectively, and have the important properties of biomedical polymers, including biocompatibility, biodegradability, non-toxicity, and mucoadhesivity with mechanical strength [7]. Chitosan is a linear heteropolysaccharide (Figure 1a) consisting of D-glucosamine and N-acetyl D-glucosamine connected with β-(1-4) glycosidic linkages [10]. 

The main groups in chitosan are amino functional groups that have a significant role in the biochemical system and electrostatic interactions in drug delivery systems, and it is soluble in a slightly acidic solution [11,12]. The gelling biopolymer pectin is derived from plants, and mainly its chain structure consists of α-(1-4) D- galacturonic acid units (Figure 1b). Its chains are interspersed by rhamnogalacturonan sections with alternate residues of rhamnose and galacturonic acid [13]. Recently, pectin-based drug delivery systems have received a great deal of attention because of their gelling capacity, biocompatibility, and low toxicity [14]. Pectin microspheres are used in drug delivery by mixing pectin with other polymers to form hybrid pectin spheres. Pectin is highly soluble in water because it has a carboxyl group; therefore, it has a higher hydrophilicity than chitosan. Adding pectin to chitosan can improve its mechanical properties and increase its hydrofility. Therefore, pectin is widely used in pharmaceutical preparations for drug delivery in the form of gels, tablets, and films. Chitosan-pectin composites could increase the microspheres’ strength so that they can control hydrophilicity and disintegration [15].

Favipiravir, the antiviral agent, is a purine nucleic acid analog (Figure 1c) that inhibits the RdRp proteins of RNA based viruses [16]. It is also used as an antiviral for Arena, Ebola, Bunya, and influenza viruses [17]. Favipiravir is an oral drug that was approved to treat COVID-19. It has shown fast viral clearance as compared to others [18].

The main purposes of this work were loading and orally releasing the Favipiravir drug under controlled conditions. A new hydrogel system was prepared by copolymerizing chitosan with freshly extracted pectin from pomegranate peels. The hydrogel was cross-linked by gluteraldehyde and sodium hexametaphosphate for chemical and physical comparison, respectively. The degree of swelling of the hydrogel microspheres was studied for both hydrogels with different cross-linkers. The hydrogel systems have been characterized by FTIR, ^1^H NMR, GC/MS, XRD, thermal, and SEM analyses. Maximum loading and efficiency of loading percentages were measured, and the loaded microspheres were allowed to release in different pH-buffered solutions, and their cumulative release percentages were calculated.

## 2. Experimental

### 2.1. Materials and Methods

Pectin (PE) was extracted from locally sourced pomegranate peels. The chitosan (CS) sample (α-chitosan, 70% DDA) was obtained from Alpha Chemika, Maharashtra, India, and was dissolved in 2% (*w*/*w*) acetic acid for purification, then filtered under pressure to remove undissolved particles of chitosan. The clear filtrate was subsequently precipitated using a 1 M NaOH solution, filtered, and dried at 25 °C under vacuum. The Favipiravir drug was supplied by the state company for the drug industry and medical appliances in Samaraa, Iraq. The glutaraldehyde (Glu) (25% *w*/*w*) solution was obtained from Thomas Baker (chemicals) Pvt. Ltd., Mumbai, India. Sodium hexameta phosphate (SHMP) and ammonium persulfate (APS) were obtained from BDH and U.K. ethanol (96% *w*/*w*), and different buffer solutions were analytical-grade chemicals (Fluka, Swiss, Buchs, Switzerland).

### 2.2. Preparation of Hydrogels

#### 2.2.1. Extraction of Pectin

Pectin was extracted from fresh pomegranate peels, which were washed with distilled water, dried, and ground. The 50 g of crushed pomegranate peels were poured inside the thimble of the soxhlet extractor, followed by 250 mL of ethanol (96% *w*/*w*) in the round bottom flask of the soxhlet apparatus [19]. After heating the ethanol to its boiling point, the solvent was allowed to recycle for two hours, passing through the crushed pomegranate peels for the removal of proteins, lipids, wax, and salt contents. Finally, the crushed pomegranate peels were taken out of the thimble of soxhlet and transferred to the reflux apparatus of a 500 mL round bottom flask, where 250 mL of distilled water was added. The solution was heated to the boiling point of water for one hour with continuous stirring, and the solution was cooled and filtered. The turbid solution was centrifuged, and the pure solution was precipitated by ethanol (96% *w*/*w*) using three times the volume of peel solution and keeping stirring extra for 30 min. Finally, the solution was kept in a refrigerator overnight. The precipitated pectin was filtered and dried in an oven at 50 °C, then grinded, and its structure was examined by FTIR, GC/MS, and ^1^H NMR.

#### 2.2.2. Preparation of Chitosan-Pectin Copolymer

A polymer solution was prepared by dissolving 1.0 g of dry and pure chitosan (CS) in 100 mL (2.0% *w*/*w*) of acetic acid prepared in distilled water, and the solution was kept with stirring for 3 h at room temperature. Similarly, 1.0 g of dry pectin (PE) was dissolved in 100 mL of distilled water and heated at 50 °C with continuous stirring for 20 min. A solution of 100 mL was prepared from previous solutions, where (1.0: 0.5M) of chitosan to pectin solution in a 250 mL beaker was mixed. The initiator solution of 5 mL (10% *w*/*w*) APS in distilled water was added slowly with continuous stirring at room temperature. The viscous chitosan/pectin solution with initiator was blown slowly through the nozzle into a beaker containing 100 mL of a neutral (5% *w*/*w*) solution of glutaraldehyde (Glu). The solution was heated at 60 °C with slow stirring, and the formed microspheres were kept under stirring for an extra 1 h. The microspheres were separated, washed several times with hot and cold water, and finally vacuum dried at 30 °C. A similar procedure has been used with only 100 mL of sodium hexametaphosphate (SHMP) at (6% *w*/*w*) concentration instead of glutaraldehyde for the preparation of chitosan/pectin microspheres.

### 2.3. Measurement of Size and Morphology of Different Microspheres

The size and morphology of prepared microspheres were measured to determine their effects on loading and release characteristics by scanning electron microscope (SEM) using TESCAN MIRA FESEM, Czech Republic, and ZEISS microscopy systems, Germany. The size and morphology of microspheres varied as the type of cross-linker was changed [20]. The shape factor (S) was calculated using size parameters for the surface characteristics of microspheres using the following equation [21].

(1) $$S=\frac{L^{2}}{4\pi A}$$

where L is the perimeter and A is the surface area of the prepared microspheres. It was shown that a value of (S) above 0.80 means the surface roughness increases progressively [20].

### 2.4. Degree of Swelling (DS) in Cross-Linked Microspheres 

The degree of swelling (DS) of the prepared microspheres with glutaraldehyde cross-linked (CS-co-PE)/Glu and with sodium hexametaphosphate cross-linked (CS-co-PE)/SHMP was measured by keeping 100 mg of dry microspheres in 20 mL of phosphate buffered solution (pH = 7). The microspheres were taken out of the buffer solution after 6 h, filtered by a fine mesh sieve of (100 mesh), left for 10 min to drain, and weighted. The swollen microspheres were then returned to the solution, and the process was repeated every 6 h until there was no change in their weight. The following equation was used for the calculation of the degree of swelling [22]:

(2) $$DS\left(\%\right)=\left(\frac{W_{t}-W_{o}}{W_{o}}\right)\times 100\%$$

where W_t_ and W_o_ are the weights of swelling microspheres after time (t) and at zero time, respectively.

### 2.5. Loading of Favipiravir on Microspheres 

The loading of Favipiravir on prepared microspheres was carried out by immersing 100 mg microspheres of (CS-co-PE)/Glu or (CS-co-PE)/SHMP in 50 mL of phosphate buffered solution (pH = 7), containing different concentrations of Favipiravir starting from 10 mg to 100 mg in the 50 mL loading solution. The loading temperature was fixed at 25 °C, and the different microspheres were kept in the loading solution for 24 h under slow stirring. The amount of Favipiravir loaded on microspheres was determined by recording the absorbance of the remaining loading solution after removing microspheres at λmax = 274 nm using the UV-1800 Shimadzu Spectrophotometer, Kyoto, Japan. The loading of Favipiravir in the microspheres was calculated as the maximum loading percentage ($L_{max}$%) using Equation (3) [21].

(3) $$L_{max\;(\%)}=\frac{weight\;of\;Favipivavir\;loading\;(mg)}{weight\;of\;microspheres\;taken\;for\;loading\;(100\;mg)}\times 100$$

Also, the loading of Favipiravir was calculated as the efficiency of loading EL_max_ (%) using Equation (4) [21].

(4) $${EL}_{max\;(\%)}=\frac{weight\;of\;Favipiravir\;loaded\;(mg)}{weight\;of\;Favipiravir\;taken\;for\;loading}\times 100$$

### 2.6. Release of Favipiravir from Loaded Microspheres

The release characteristics of chitosan/pectin microspheres of both cross-linked types were determined by immersing 100 mg of Favipiravir loaded microspheres in a 20 mL buffered solution of different pH at 37 °C where the release behavior of the Favipiravir loaded microspheres has been done at pH = 1.3, pH = 7.4, and pH = 9.4, which simulate the pH of gastric fluid, plasma blood fluid, and intestinal fluid in the human body, respectively [23]. The amount of Favipiravir released in the media was fixed by recording the absorbance at λmax = 274 nm of the solution sample withdrawn from the release media and replaced with the same quantities of native solution. The release of Favipiravir was given as a controlled release percentage (CRmax%) using Equation (5) [24];

(5) $$\mathrm{Controlled}\;\mathrm{release}\;(\mathrm{CRmax})\;\%=\sum (\frac{W_{t}}{W_{o}}\times {100)}_{constant}$$

and as burst release percentage (BRmax%) using Equation (6);

(6) $$\mathrm{Burst}\;\mathrm{release}\;(\mathrm{BRmax})\;\%=\sum (\frac{W_{t}}{W_{o}}\times {100)}_{variable}$$

where $[\frac{Wt}{W_{o}}$ $\times \;{100]}_{t}$ is a variable amount for burst release and a constant amount for controlled release at 37 °C and for a fixed time interval of 6 h.

The overall release was calculated as the cumulative release percentage (Rcum%) using Equation (7) for a fixed time interval of 6 h.

(7) $$\mathrm{Cumulative}\;\mathrm{release}\;(\mathrm{Rcum})\;\%=\frac{W_{t}}{W_{o}}\times 100$$

where W_t_ is the cumulative amount of Favipiravir released at time (t) and $W_{o}$ is the total amount of Favipiravir released [23].

## 3. Results and Discussion

Natural polymers like polysaccharides, which are biocompatible materials used in drug release systems, have the ability to encapsulate drugs and release them in a sustained and controlled manner [24]. The polymeric systems’ physicochemical properties can play a significant role in controlling drug release from the systems. Moreover, copolymerization of chitosan, the cationic polysaccharide, with pectin, the anionic hetropolysaccharide, produces hydrogel with both functional groups helping the hydrogel swelling to a wide range in different pH media. The high degree of swelling in hydrogels can be loaded with chronic drugs or those that have harmful effects on the digestive system. The loaded drug hydrogels have shown long-term release under controlled conditions.

**Figure 2:**
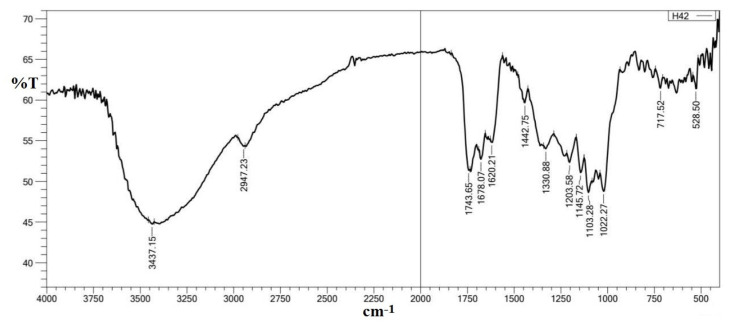
FTIR spectrum of polysaccharide extracted from pomegranate peels.

Pectin was extracted from pomegranate peels, and before copolymerizing with chitosan, its structure was characterized by FTIR using an 8400 Shimadzu spectrophotometer, Japan, with (400–4000 cm^−1^). The following major peaks of the extracted pectin (Figure 2) were studied, where the absorption frequency at 3437 cm^−1^ belongs to the hydroxyl group ν (O-H)str. The band at 2947 cm^−1^ represents the methine group ν (C-H)str in pectin. The absorption frequency at 1744 cm^−1^ is attributed to the carbonyl group ν (C=O)str of the methyl ester group (COOCH_3_), and that at 1678 cm^−1^ belongs to ν (C=O)str of undissociated carboxylic acid (COOH) [25]. The absorption band at 1620 cm^−1^ represents asymmetric stretching vibrations belonging to (C=O) of the carboxylate ion (COO^−^). The absorption bands that appeared at 1443 cm^−1^ and 1331 cm^−1^ (Figure 1) belong to (-CH_2_) scissoring and (-OH) bending vibrations, respectively. Finally, those bands appearing at 1146 cm^−1^ and 1022 cm^−1^ represent (-OH) the secondary alcohol in the aliphatic cycle and stretching of the methoxy group, respectively.

**Figure 3:**
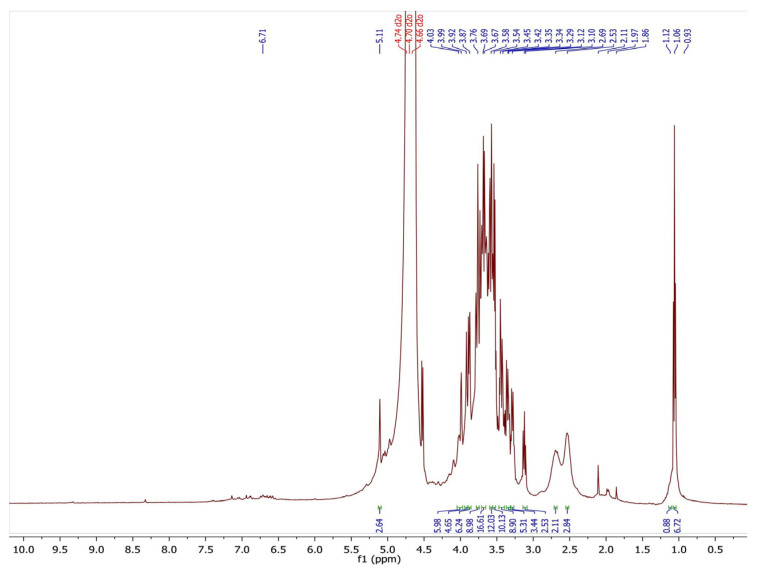
^1^H NMR spectrum of extracted polysaccharide from pomegranate peels.

The ^1^H NMR spectroscopy of the extracted pectin was studied by a Varian Inova spectrophotometer at 500 MHz in Palo Alto, CA, USA, using deuterated water as a solvent. The ^1^H NMR spectrum (Figure 3) shows resonance (3H,m) at (1.06 and 1.12) ppm, which represents the methyl (CH_3_) group of rhamnose. The resonance (3H,w) at (1.86 and 1.97) ppm represents the acetyl group located at 2- and 3-O- galacturonic acid, respectively whereas the resonance (3H,m) observed at 3.76 ppm (Figure 2) belongs to the methoxy (CH_3_O-) group of the galacturonic acid esterification unit. The following resonances at (3.69, 3.8, 3.92, 4.03, and 5.11) ppm represent the protons of galacturonic acid units [26,27].

**Figure 4:**
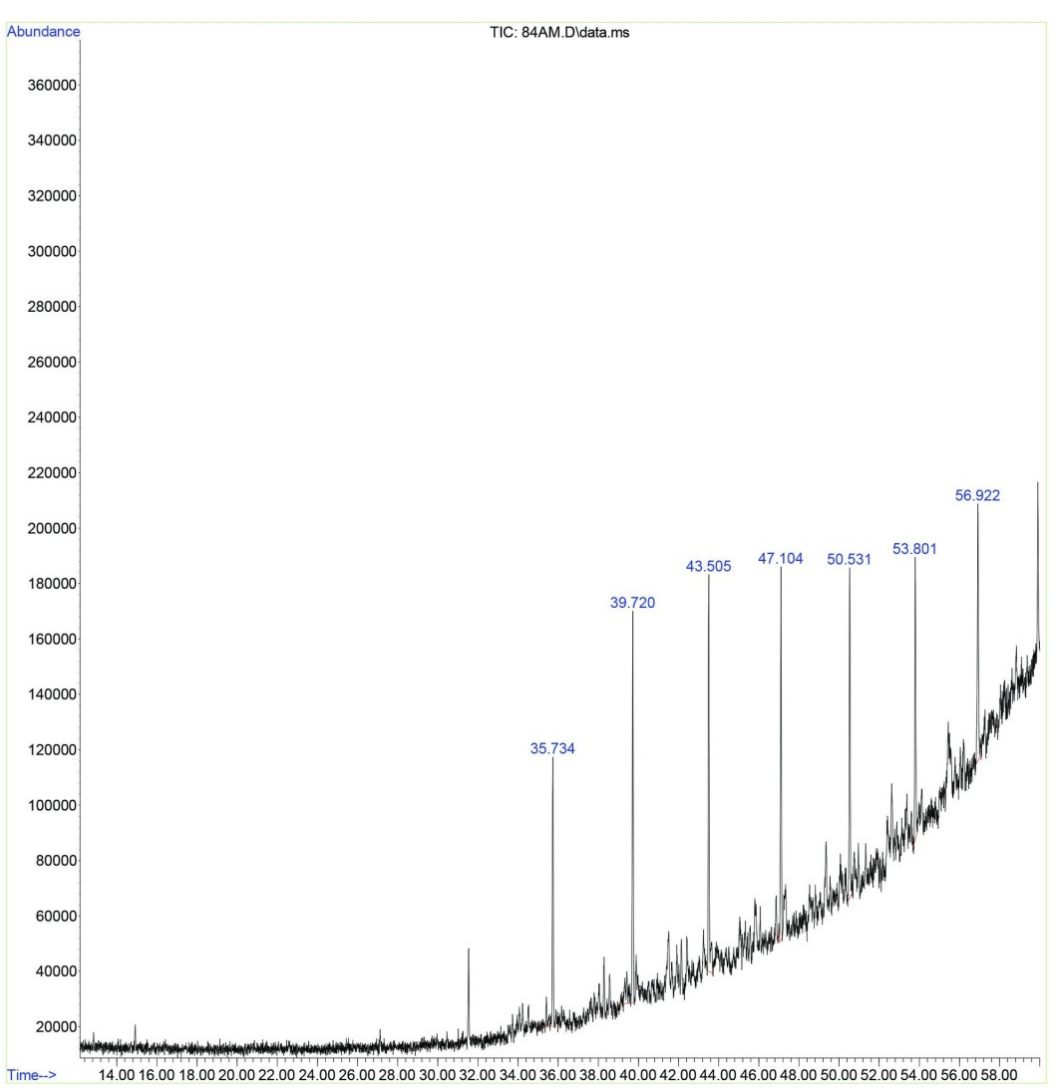
GC/MS spectrum of extracted polysaccharide from pomegranate peels.

The GC/MS analysis was also used for the characterization of extracted pectin, where the analysis was carried out by Agilent 6890/5973 inert GC/MSD; Agilent Technologies; Palo Alto, CA, USA. The extracted pectin from pomegranate peels has shown many neutral sugars distributed gradually (Figure 4). They start with glucose, followed by mannose, arabinose, xylose, galactose, rhamnose, and others. In addition, uronic acid with low and high amounts of fiber is present (Figure 4). The presence of galactose and rhamnose, the main components of pectin, means the extracted polysaccharide from pomegranate peels is mainly pectin, along with other natural materials such as wax, proteins, and some salts [28,29].

### 3.1. Studies of the Prepared Hydrogels

**Figure 5:**
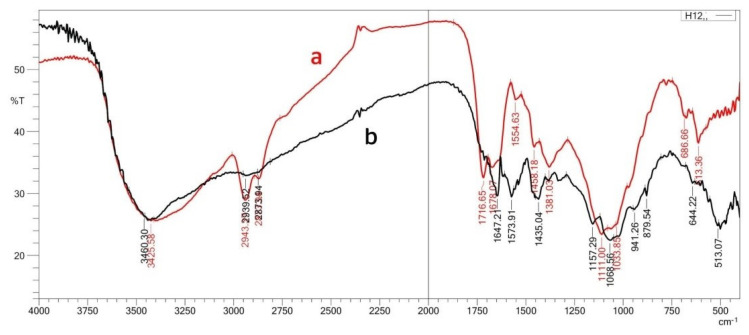
FTIR spectra of (a) (CS-co-PE)/Glu and (b) (CS-co-PE)/SHMP microspheres.

Hydrogels cross-linked differently were prepared by copolymerizing chitosan with freshly extracted pectin. Generally, the prepared hydrogels were cross-linked chemically by glutaraldehyde (CS-co-PE)/Glu and physically by sodium hexametaphosphate (CS-co-PE)/SHMP. The hydrogels were characterized, and their FTIR spectra (Figure 5 and Table 1) show an absorption frequency of 3426 cm^−1^ represents the hydroxyl group of both chitosan and pectin. The band at 1717 cm^−1^ belongs to the carbonyl group of methyl ester and undissociated carboxylic acid. Whereas, the band at 1678 cm^−1^ representing the (amide-I) of chitosan beside the carbonyl group of glutaraldehyde and pectin. The absorption frequency at 1555 cm^−1^ belongs to (amide-II) of chitosan, while the band at 1640 cm^−1^ (Figure 5a and Table 1) belongs to the imine bonding (C=N) formed due to the interactions between the amine group of chitosan and the aldehyde group of glutaraldehyde [25]. The peaks of (CS-co-PE)/SHMP are almost similar to those of (CS-co-PE)/Glu except those at 1157 cm^−1^ and 1069 cm^−1^ (Figure 5b and Table 1), which represent the P-O-P connections in sodium hexametaphosphate [30].

The ^1^H NMR spectroscopy of (CS-co-PE)/Glu hydrogel (Table 2) has shown the resonance of protons of the main groups in the hydrogel composite. The resonances of (2H,m) at (1.0–1.7) ppm (Table 2) represent the methylene (CH_2_) groups of glutaraldehyde. The signals of (3H,w) at 1.84 and 1.96 ppm belong to the acetyl groups of galacturonic acid in pectin. The methoxy group of the galacturonic acid esterification unit in pectin gives a signal at 3.72 ppm (Table 2). While chitosan produce the main resonance at 1.8 ppm of (3H,s), which represents the acetyl group of N-acetylglucosamine, the signal of (2H,m) at 3.0 ppm belongs to the D- glucosamine fraction. The resonance at (3.0–3.8) ppm (Table 2) represents the cross-linking of glucosamine groups with glutaraldehyde [31].

**Figure 6:**
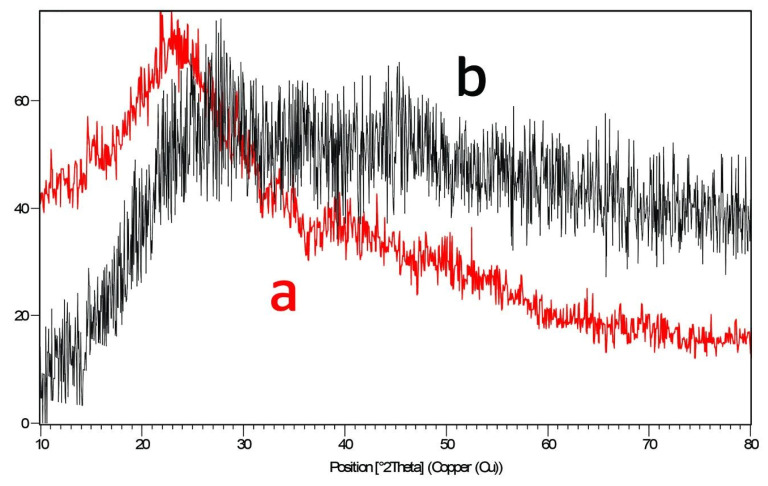
XRD Pattern of (a) (CS-co-PE)/Glu and (b) (CS-co-PE)/SHMP hydrogels.

The XRD pattern of both hydrogels (Figure 6) has shown that the compounds are amorphous in their nature with low crystalline structures, and the broad maxima starting from 20°θ to 26°θ in (CS-co-PE)/Glu belong to the polysaccharides chitosan and pectin (Figure 6a), which was shifted a little bit to 28°θ (Figure 6b) under the effect of the crystalline nature of sodium hexametaphosphate in (CS-co-PE)/SHMP [32].

The thermal studies of the prepared hydrogels using SDT Q600 V20.9 Build 20 show the hydrogels are thermally stable almost up to 200 °C. Where the TGA of (CS-co-PE)/Glu hydrogel (Table 3) has been shown at the initial decomposition temperature (IDT), the weight loss% was 2.0% at 72 °C, representing the weight loss of the free water. Moreover, at the final decomposition temperature (FDT), the weight loss % was 52.0% at 382 °C and 32.0% at 277 °C, representing the maximum decomposition temperature (Tmax) whereas 60.0% weight loss at 450 °C represents the crystalline decomposition temperature (Tcr) (Table 3). The DSC thermogram of (CS-co-PE)/Glu hydrogel (Table 3) shows the (Tg) of the hydrogel is at 42 °C and has a heat of fusion (∆Hf) at 240 °C of (+389 J/g) with endothermic behavior. Similarly, the thermogram of (CS-co-PE)/SHMP hydrogel has shown a weight loss% of 2.9% at 92 °C, representing IDT, and 46.0% at 398 °C, representing FDT. The weight loss% is reaching 28.5% at 290 °C, representing Tmax, whereas the weight loss% is 56.8% at 458 °C, representing Tcr. The DSC thermogram of (CS-co-PE)/SHMP hydrogel has shown thermal data, especially the glass transition temperature Tg = 51 °C, which is higher than Tg = 42 °C of (CS-co-PE)/Glu hydrogel (Table 3) because of its higher crystallinity, which belongs to its ionic cross-linker (SHMP). Therefore, it gives the impression that (CS-co-PE)/SHMP hydrogel is thermally more stable.

**Figure 7:**
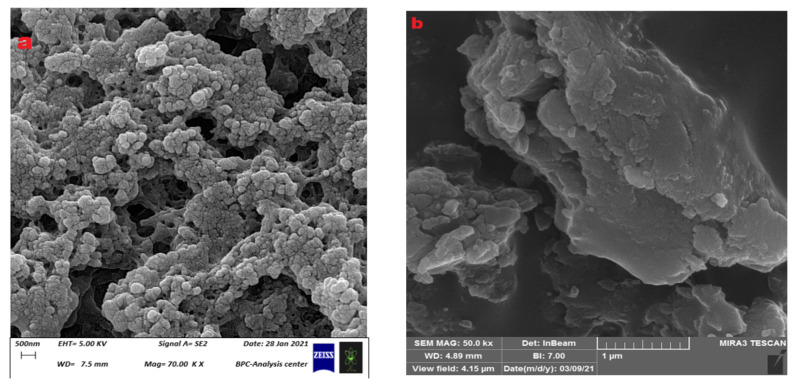
SEM images of (**a**) (CS-co-PE)/Glu and (**b**) (CS-co-PE)/SHMP.

The SEM images of the prepared hydrogels were studied (Figure 7), and the SEM image of CS-co-PE/Glu hydrogel (Figure 7a) shows clusters of microspheres with non-uniform surfaces accumulated as irregular agglomerates showing a non-crystalline, elastic nature interspersed with holes and folds. Those properties give the CS-co-PE/Glu hydrogel a high ability for loading and delivering drugs. The SEM image of CS-co-PE/SHMP hydrogel (Figure 7b) has shown compact microspheres with small sizes that have almost the same properties as CS-co-PE/Glu hydrogel microspheres, only they accumulate more in a cluster form.

Finally, the surface area of the prepared hydrogel particles was studied using the BET technique, type BELSORP MINI II, surface area, and porosimetry analyzer (Osaka, Japan). The BET Plot has shown that the specific BET area of CS-co-PE/Glu hydrogel particles is (2.261 m^2^/g) with a total pore volume (0.0059 cm^3^/g) (Table 4), while CS-co-PE/SHMP hydrogel particles have a specific BET area (334.88 m^2^/g) and a total pore volume (0.918 cm^3^/g) (Table 4). The BJH plot of (CS-co-PE)/Glu hydrogel particles gives the pore area (1.21 nm) and specific surface area (5.0978 m^2^/g) (Table 4) where the pore area of (CS-co-PE)/SHMP hydrogel particles is (4.61 nm) and its specific surface area is (420.24 m^2^/g) (Table 4). The BET measurement data shows that the (CS-co-PE)/SHMP hydrogel particles have a more specific surface area than the (CS-co-PE)/Glu hydrogel particles.

### 3.2. Surface Measurement of Microspheres and Their Physical Characteristics

**Figure 8:**
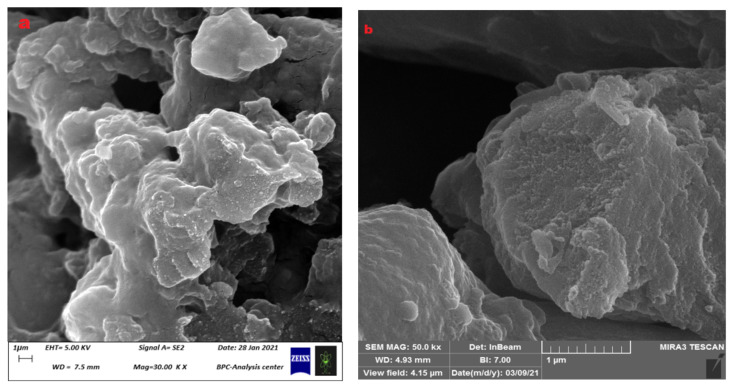
SEM image of (**a**) (CS-co-PE)/Glu, (**b**) (CS-co-PE)/SHMP hydrogels.

The type of cross-linking has shown significant effects on the morphology of the hydrogel microspheres [20,33] from studying the SEM images of microspheres cross-linked with glutaraldehyde (Glu) (Figure 8a) and sodium hexametaphosphate (SHMP) (Figure 8b). Those microspheres cross-linked with Glu were large in size (74.97 μm) with a rough surface (Figure 8a), while those cross-linked with SHMP had a smaller size (20.47 μm) and a smoother surface (Figure 8b). Another effect on the surface roughness of microspheres has been shown through changes in the type of cross-link, where the change appears clearly in the shape factor (S) of microspheres that is calculated by Equation (1). With a high value of shape factor (S), greater than 0.80, the surface of microspheres tends to be rough, while it becomes smooth when S is equal to or lower than 0.80. The microspheres cross-linked by Glu have shown a high value of S (0.852), which means a high degree of roughness (Figure 8a) whereas those microspheres cross-linked with SHMP show a low value of S (0.748); therefore, the microspheres have smooth surfaces (Figure 8b).

### 3.3. Degree of Swelling

**Figure 9:**
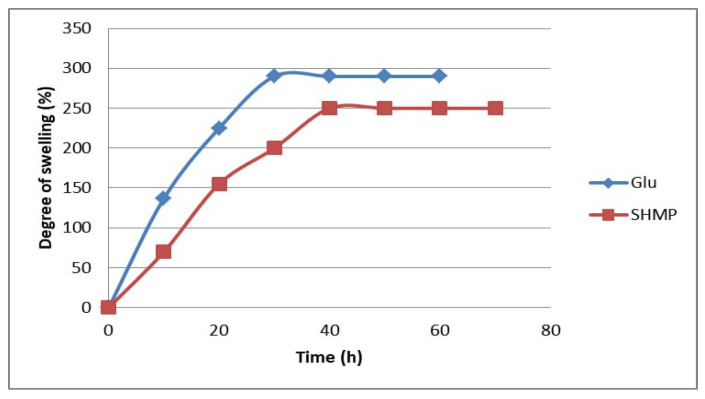
Degree of swelling of (CS-co-PE)/Glu and (CS-co-PE)/SHMP microspheres in pH7.

The degree of swelling in hydrogel microspheres could control the loading of drugs and their release behavior; hence, the swelling characters of (CS-co-PE)/Glu and (CS-co-PE)/SHMP hydrogel microspheres are given in (Figure 9). The microspheres cross-linked with Glu reveal a maximum degree of swelling of 290%, while those cross-linked with SHMP have a maximum degree of swelling of 250%. In general, the morphology of microspheres from the SEM micrographs has shown that microspheres cross-linked with Glu have a rough surface (Figure 8a) with a high value of shape factor (S) greater than 0.80, which means easier penetration of phosphate buffered solution pH = 7 into the polymer chains and finally increases the degree of swelling whereas microspheres cross-linked with SHMP have a smooth surface and lower S with a lower degree of swelling (Figure 9).

### 3.4. Loading of Favipiravir on Prepared Microspheres

**Figure 10:**
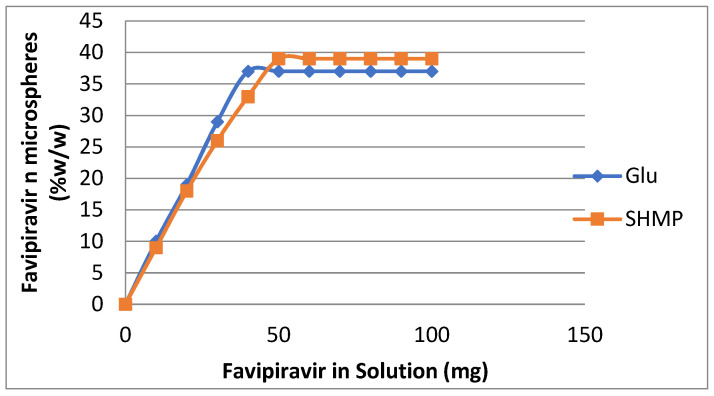
Loading of Favipiravir on (CS-co-PE) microspheres cross-linked with Glu and SHMP. Loading time = 24 h, loading media = 100 mg microspheres in a 50 mL phosphate buffered solution of Favipiravir pH = 7, T = 25 °C.

The differences in cross-linking of microspheres have significantly influenced many physico-chemical properties of the hydrogel microspheres, including their loading behavior with Favipiravir. The loading process of Favipiravir in microspheres was done, where 100 mg microspheres were kept in a 50 mL solution prepared from Favipiravir dissolved in a buffered solution of pH = 7 under slow stirring for 24 h. To study the effect of the initial concentration of Favipiravir, the concentration of Favipiravir was varied from 10 mg to 100 mg in a 50 mL loading solution. The microspheres cross-linked with Glu (Table 5, Figure 10) resulted in a maximum loading (L_max_) of 35 mg per 100 mg microsphere. Whereas the microspheres are cross-linked with SHMP (Table 5, Figure 10), their maximum loading (L_max_) has increased to 37 mg per 100 mg microspheres. The increasing trend in maximum loading (L_max_) for Favipiravir in SHMP cress-linked microspheres (37 mg Favipiravir per 100 mg microspheres) was due to highly electrostatic interactions between the water-soluble Favipiravir at pH = 7 [34] and the highly ionic salt sodium hexametaphosphate anions. Added to that, the dispersal holes and folds on their surfaces, beside the SHMP microspheres, are present in clusters, which significantly increase the retention capacity of SHMP microspheres in comparison with the non-ionic Glu microspheres.

### 3.5. Release of Favipiravir from Loaded Microspheres

**Figure 11:**
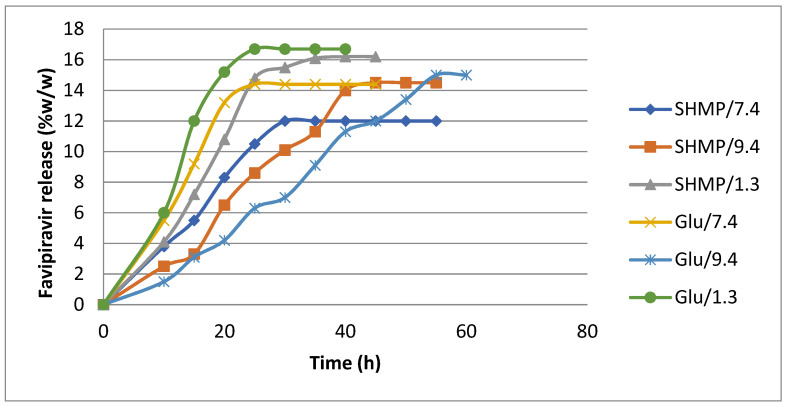
Effect of solution pH on the release percentage of Favipiravir from Glu and SHMP cross-linked (CS-co-PE) microspheres. Release media = 100 mg loaded microspheres in 20 mL buffered solution (pH = 7.4, pH = 9.4, and pH = 1.3), at T = 37 °C.

The release profile of Favipiravir has indicated that the release of the drug is dependent on the type of cross-linking and on the pH of the release medium where the pH of the release medium has controlled the drug release pattern of microspheres by changing their degree of swelling due to variations in electrostatic interactions between microspheres and their ionic cross-linkers like sodium hexametaphosphate anions. The release pattern of Favipiravir from microspheres at different pHs was studied (Table 5, Figure 11), and Favipiravir was released first with a burst trend (%R_B_), where the amount of released drug increased in a fixed interval of time (Table 5, Figure 11). Moreover, the polymer chains in microspheres were relaxed, and the degree of swelling continued to increase. With microspheres, structural changes have taken place. Accordingly, because the drug was released in bursts and was not constant, this type of release is not useful for sustainable drug release. After the microspheres’ structure reaches equilibrium, they start releasing drugs in a constant manner within a fixed interval of time. This step of drug release with a controlled trend (%Rc) has shown (Table 5, Figure 11) dependence on the release media and types of microsphere cross-linkers. The trend in burst release of Favipiravir from Glu microspheres has shown an increase from 9.8 mg in a release solution of pH = 7.4 to 23.8 mg in pH = 9.4. At high pH, the Glu microspheres reach their high structural relaxation, whereas in an acidic release solution of pH = 1.3, the microspheres compact the same as those in pH = 7.4 and reach their controlled release with minimum loss. The Glu microspheres give a controlled release trend different from the burst release step, where they release in controlled fashion for 30 h in a release solution of pH = 7.4, and the released amount of drug represents 72% *w*/*w* of their load of Favipiravir (Table 5, Figure 11). Similarly, the Glu microspheres released 66.8% *w*/*w* of their loaded Favipiravir in a controlled manner for 24 h in a release solution of pH = 1.3, meaning the microspheres swelled to a moderate degree in an acidic medium. The release at pH = 9.4 was mostly in burst steps, and only 48.6% *w*/*w* of the loaded Favipiravir was released in controlled steps and only for 18 h.

On the other hand, the SHMP microspheres have shown almost the same trend as the Glu microspheres. The Favipiravir loaded microspheres were released at 72% *w*/*w* of their loaded Favipiravir for 36 h in a release solution of pH = 7.4 (Table 5, Figure 11). However, the main difference between the two systems is the controlled release time, which is 36 h for SHMP microspheres and 30 h for Glu microspheres (Table 5, Figure 11). In addition, the release of Favipiravir from SHMP microspheres in release media of pH = 9.4 was improved under controlled conditions, while it seemed not to be as good in pH = 1.3 acidic release media. This may be due to the electrostatic attraction between chitosan and its cross-linker SHMP ions, which hinders the release of Favipiravir.

**Figure 12:**
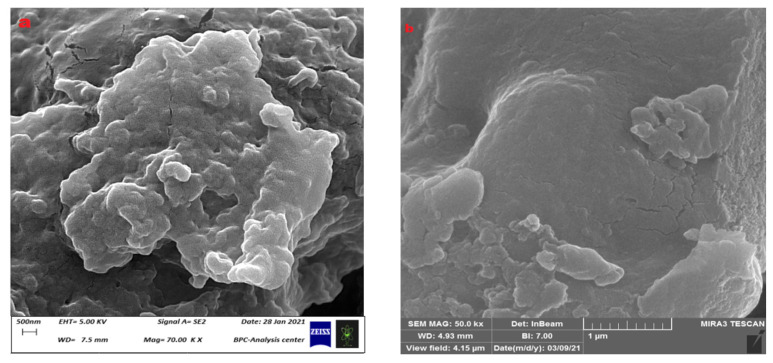
SEM images of (**a**) (CS-co-PE)/Glu and (**b**) (CS-co-PE)/SHMP microspheres after release.

The SEM images of the released microspheres (Figure 12a,b) show a cracked form in their structure after the release of the loaded Favipiravir into the release medium. Accordingly, the SEM image of Glu microspheres (Figure 12a) has shown that the microspheres after release in pH = 7.4 media still have an integrated structure with some cracks on their surface permeated with a few holes, which may be due to the exit of Favipiravir. Moreover, the SHMP microspheres have shown a SEM image after release (Figure 12b) that is almost identical to that of the Glu microspheres, except some microspheres are broken and their pieces are scattered, which may be due to their brittle structure.

## 4. Conclusions

Chitosan was copolymerized with pectin, where the latter was extracted from fresh pomegranate peels and characterized for its structure. The prepared copolymers were cross-linked physically by SHMP, and chemically, the microspheres were cross-linked by Glu. The formed microspheres were characterized for their structures, crystallinity, thermal behaviors, surface morphologies, surface area, and degree of swelling. Types of cross-linking of microspheres have shown significant effects on their surface morphology; SHMP cross-linked microspheres appeared small in size with a smooth surface, while Glu cross-linked microspheres had a larger size with a rough surface. The loading of chitosan/pectin microspheres with Favipiravir and their release characteristics were evaluated as a function of physical and chemical cross-linking and varying the solution pH of Favipiravir release media. The depression in the degree of swelling of SHMP cross-linked microspheres shows the compactness in their form structure in comparison with those of Glu microspheres, as the shape factor (S) measurements proved. The prepared microspheres showed after 24 h inside a pH7 buffered solution of Favipiravir a maximum loading of 37% (*w*/*w*) for SHMP microspheres and 35% (*w*/*w*) for Glu microspheres. The release characteristics of leaded microspheres have shown differences when varying the pH of the release solution from 1.3 to 7.4 and then to 9.4, especially in the case of SHMP microspheres, due to the strength of electrostatic interactions between sodium hexametaphosphate anions and the ionized chitosan/pectin molecules, which controlled the release of Favipiravir in a better way. The SHMP microspheres in pH = 7.4 release media have shown a better controlled pattern for releasing Favipiravir, where the media ions could help for more interactions. Whereas, the Glu microspheres system has shown controlled release of Favipiravir better at pH = 1.3 than at pH = 7.4, which may provide more protons for electrostatic interactions between chains of chitosan and pectin molecules.

## Figures and Tables

**Table 1:** FTIR major functional groups of Glu and SHMP cross-linked microspheres with absorption frequencies.

FTIR Characteristic Functional Groups								
Sample	(O-H)str	(C=O)str	(C=O)str Amide-I	(N-H)str Amide-II	(-C=N-)str	(C=O)str symm.	(N-H)band	(p-o-p)
	Wave Numbers *v*/cm^−1^							
CS-co-PE/Glu	3426	1717	1678	1555	1640	1381	1111	……
CS-co-PE/SHMP	3460	1724	1647	1574	1622	1388	1157	1157 1069

**Table 2:** ^1^H NMR chemical shift of the main protons of CS-co-PE/Glu hydrogel.

Sample	Chemical Shift σ/ppm	Description of Proton
CS-co-PE/Glu	1.0–1.7	(CH_2_) groups of gluteraldehyde.
	1.04–1.1	(CH_3_) groups of rhamnose.
	1.84–1.96	Acetyl groups of pectin.
	3.72–3.8	Methoxy group of pectin & D-glucopyranose of chitosan.
	3.5–4.0	Protons of glucopyranose ring in chitosan.
	3.0–3.8	Cross-linking of glucosamine groups with gluteraldehyde.

**Table 3:** TGA and DSC thermal data of the Glu and SHMP cross-linked microspheres.

Sample	TGA Weight Loss (%)				DSC (w/g)	
	IDT/°C	FDT/°C	T_max/_°C	T_cr_/°C	Tg/°C	∆ Hf (J/g)
(CS-co-PE)/Glu	2.0% 72 °C	52% 382 °C	32.0% 277 °C	60.0% 450 °C	42 °C	+389 J/g 240 °C
(CS-co-PE)/SHMP	2.9% 92 °C	46% 398 °C	28.5% 290 °C	56.8% 458 °C	51 °C	+468 J/g 251 °C

**Table 4:** BET measurements of microspheres and their specific surface area.

Plot Type	Plot Data	CS-co-PE/Glu	CS-co-PE/SHMP
		Adsorption Branch	
BET Plot	V_m_	0.5184 [cm(STP)g^−1^]	76.94 [cm^3^(STP)g^−1^]
	a_s_, BET	2.2561 [m^2^g^−1^]	334.88 [m^2^g^−1^]
	Total pore volume (P/P_o_ = 0.990)	0.0059719 [cm^3^g^−1^]	0.9177 [cm^3^g^−1^]
	Mean pore diameter	10.588 [nm]	10.961 [nm]
BJH Plote	V_p_	0.0070665 [cm^3^g^−1^]	0.9339 [cm^3^g^−1^]
	r_p_, peak (Area)	1.21 [nm]	4.61 [nm]
	a_p_	5.0978 [m^2^g^−1^]	420.24 [m^2^g^−1^]

**Table 5:** Loading and release characteristics of (CS-co-PE) microspheres cross-linked with Glu and SHMP.

Microsphere Sample	Max. Loading L_max_ (mg)	Release Solution pH	Burst Release (mg)	Controlled Release (mg)	Controlled Release Time (h)
CS-co-PE/Glu	35	1.3	11.6	23.4	24
		7.4	9.8	25.2	30
		9.4	23.8	11.2	12
CS-co-PE/SHMP	37	1.3	19.2	17.8	18
		7.4	10.4	26.6	36
		9.4	20.9	16.1	18
